# Medication management in long-term care: using evidence generated from real-world data to effect policy change in the Australian setting

**DOI:** 10.1093/aje/kwae136

**Published:** 2024-06-18

**Authors:** Janet K Sluggett, Maria C Inacio, Gillian E Caughey

**Affiliations:** University of South Australia, UniSA Allied Health and Human Performance, Adelaide, South Australia, Australia; Registry of Senior Australians, South Australian Health and Medical Research Institute, Adelaide, South Australia, Australia; University of South Australia, UniSA Allied Health and Human Performance, Adelaide, South Australia, Australia; Registry of Senior Australians, South Australian Health and Medical Research Institute, Adelaide, South Australia, Australia; University of South Australia, UniSA Allied Health and Human Performance, Adelaide, South Australia, Australia; Registry of Senior Australians, South Australian Health and Medical Research Institute, Adelaide, South Australia, Australia

**Keywords:** pharmacoepidemiology, medication review, long-term care, nursing homes, Australia

## Abstract

Older individuals residing in long-term care facilities (LTCFs) are often living with multimorbidity and exposed to polypharmacy, and many experience medication-related problems. Because randomized controlled trials seldom include individuals in LTCFs, pharmacoepidemiological studies using real-world data are essential sources of new knowledge on the utilization, safety, and effectiveness of pharmacotherapies and related health outcomes in this population. In this commentary, we discuss recent pharmacoepidemiological research undertaken to support the investigations and recommendations of a landmark public inquiry into the quality and safety of care provided in the approximately 3000 Australian LTCFs that house more than 240 000 residents annually, which informed subsequent national medication-related policy reforms. Suitable sources of real-world data for pharmacoepidemiological studies in long-term care cohorts and methodological considerations are also discussed.

This article is part of a Special Collection on Pharmacoepidemiology.

## Introduction

Medications are largely beneficial but are also a source of considerable unintended harm for older people, particularly for those residing in long-term care facilities (LTCFs), which provide personal care, nursing care and accommodation for individuals who are no longer able to remain at home.^1^ Residents of LTCFs (also known as nursing homes, care homes, or residential aged care facilities) are increasingly older and frailer upon entering care and are commonly exposed to polypharmacy and complex medication regimens. For example, 6 of every 10 new residents of Australian LTCFs take at least 1 high-risk medication (eg, anticoagulants, opioids, insulin)^2^^,^^3^ and virtually all (>95%) experience at least 1 medication-related problem.^4^ Medication use is prone to more errors than nearly any other type of care, with medication errors costing an estimated USD 42 billion annually worldwide.^5^ Hence, reducing medication-induced harm among older people is an international priority.^5^^-^^7^

Strategies to optimize medication safety and effectiveness in LTCFs can occur at the individual (eg, comprehensive medication reviews), facility or organization (eg, workforce education, incident reporting systems), or population (eg, quality and safety monitoring programs, government regulation of medication subsidies) level. Despite adoption of these types of multitiered strategies in many countries, medication-related problems still persist. For example, little to no change in the prevalence of use of antipsychotics, opioids, other sedating medications, and antibiotics were observed in Australian LTCFs between 2015 and 2019^8^ and increased sedative use has been observed in Canadian LTCFs after the onset of the COVID-19 pandemic.^9^

Randomized controlled trials (RCTs) of medication effectiveness and safety are seldom undertaken among older people living in LTCFs, who are the focus of only 2% of aging research.^10^^-^^12^ Of the 39 RCTs conducted in Australian LTCFs and published during 2000-2018, only 4 trials focused on an intervention involving pharmacotherapy for management of changed behaviors among residents living with dementia (ie, risperidone, estrogen patches, lavender oil) or falls (ie, vitamin D supplementation).^12^ Hence, evidence from existing RCTs is not sufficient to comprehensively ascertain medication safety and effectiveness in LTCF populations, who are at risk of medication-related adverse events due to polypharmacy and age-related changes in pharmacokinetics and pharmacodynamics.^11^^,^^13^ Pharmacoepidemiological studies using real-world data are, therefore, essential sources of new knowledge on the utilization, safety, and effectiveness of pharmacotherapies and related health outcomes in this population.

## Sources of real-world data for pharmacoepidemiological studies in long-term care

Real-world data are defined as “data relating to patient health status and/or the delivery of health care routinely collected from a variety of sources.”^14^ Commonly used sources of population-based real-world data suitable for use in pharmacoepidemiological studies are briefly described below. These data sources might include aggregate, cross-sectional, or longitudinal medication data, and could exist as stand-alone sources or be part of larger, integrated, multisetting data resources (eg, hospitalization records, LTCF service records, vital records).

• Medication data extracted from electronic medication management (eMM) systems or paper-based medication administration charts are considered the gold standard for comprehensive and timely medication exposure ascertainment in LTCFs.^15^ Yet, interoperability between eMM and other care provider systems, and data coding across different eMM systems can vary. Data collection from paper-based charts can be time consuming and requires standardized entry systems.

• Pharmaceutical claims data, often used to ascertain medication exposure in LTCFs,^16^^-^^18^ are often collected and coded in a standardized way. Limitations with some pharmaceutical claims data sets can include the inability to capture medication dose, treatment indication, and/or nonsubsidized or hospital inpatient medication use.

• Patient records maintained by general medical practitioners (GPs)^19^ provide data on diagnoses, treatment indications, and prescriptions (including nonsubsidized prescriptions) issued but may not reflect current medication use; treatments may have been ceased and services may be accessed from multiple GPs.

• Information collected via standardized data entry systems during medication reviews or dose administration aids have been successfully analyzed.^20^^,^^21^ Limitations can include the incomplete capture of the administration of “when needed” medications.

• Medication complaints or incident report data sets^22^^,^^23^ may comprehensively describe the history and outcomes of a specific medication-related problem but may not be individually linked or include comprehensive information about all medications taken by an individual.

Evidence from high-quality pharmacoepidemiological studies is valuable for guiding treatment discussions with residents of LTCFs and their family members, as well as informing quality improvement and aged care policy decision-making. The following case study demonstrates how we have generated evidence using real-world data from a national aged care and health data platform to inform medication management policy and practices in Australian LTCFs.

## Using real-world data to produce evidence for policy and practice change in long-term care: an Australian example

In Australia, the culmination of multiple state and federal investigations over 20 years, together with mounting public concern about care provision in LTCFs, led the Australian Government to announce a Royal Commission into Aged Care Quality and Safety in 2018. Investigating the quality and safety of medication management practices was included in the terms of reference for this public inquiry.^24^ Concerns about medication management were described in one-third of all online submissions,^24^ and the initial public hearings of the Royal Commission had a priority focus on chemical restraint.

Pharmacoepidemiological research on the use of psychotropic medications conducted using linked health and aged care data from the Registry of Senior Australians (ROSA; established in 2017)^25^ was used to support the work of this landmark inquiry through commissioned reports and expert witness testimony. ROSA’s Historical Cohort is a national resource that contains comprehensive, deidentified, integrated data on the use of aged care services, medication, health services, social welfare, and mortality information for all older individuals accessing government-subsidized aged care services from 2002 onward. The work provided to the Royal Commission reported a substantial increase in antipsychotic use among people with dementia in the lead-up to and after LTCF admission (from 8% in the 9-12 months before admission to 33% in the 0-3 months after entering care).^26^ It also demonstrated an association between antipsychotic initiation on LTCF entry and a higher risk of death compared with individuals who did not commence an antipsychotic.^27^

In response to these findings, an urgent recommendation to reduce the overreliance on chemical restraint in LTCFs was made in the Royal Commission Interim Report.^24^ The Australian Government quickly responded by announcing additional funding for dementia care training and comprehensive medication review programs, and restrictions on risperidone use for managing the behavioral and psychological symptoms of dementia.^28^^,^^29^ Guidelines to inform medication management and psychotropic medication use in Australian LTCFs have since been released.^30^^,^^31^

One strategy recommended in the Interim Report to limit psychotropic medication use was to improve access to government-subsidized comprehensive medication reviews (known as residential medication management reviews [RMMRs]) and to monitor the quality and consistency of these reviews.^32^ These reviews, which are recommended for all residents on LTCF entry and when clinical circumstances change, are provided by pharmacists in collaboration with GPs and LTCF providers to facilitate best-possible medication use. Similar programs exist in other countries, such as the United States.^32^ Evidence presented at the Royal Commission showed that information on long-term health outcomes for individuals receiving RMMRs was lacking. The Royal Commission Interim Report also recommended the organizations responsible for Australia’s community pharmacy agreement, a recurring 5-year agreement with the government to fund pharmacist-led services,^33^ to review the effectiveness of the existing RMMR program. Working with an industry body owned by the community pharmacy agreement signatories, we undertook a program of work using real-world data to examine RMMR provision after LTCF entry, changes in medication use after an RMMR, associations between RMMR provision and health outcomes, and associations between changes to RMMR program funding rules and service utilization.^2^^,^^18^^,^^34^^-^^36^ This work, which has now both informed and addressed key recommendations arising from the Royal Commission, provided timely new evidence of the effectiveness of RMMRs on improving mortality risk.^35^ In July 2024, the RMMR program was further enhanced by a new aged care onsite pharmacist program that enables pharmacists to have a greater onsite presence in LTCFs.^37^

The Royal Commission into Aged Care Quality and Safety’s 148 recommendations for sector reform were delivered in its final report (March 2021).^37^ This included recommendations to support medication safety and effectiveness by embedding pharmacists in LTCFs, requiring use of electronic medication management systems, and expanding quality indicator monitoring.^37^ Pharmacoepidemiological investigations using real-world data are now being used to inform decision-makers and respond to the subsequent aged care reforms being implemented by the Australian Government. For example, ROSA has developed a LTCF Outcome Monitoring System comprising 12 risk-adjusted quality indicators, of which 5 are medication-related (ie, antibiotic use, antipsychotic use, opioid use, high sedative load, and medication-related hospitalizations).^8^^,^^38^ Using ROSA’s Prospective Cohort, which includes identifiers for organizations delivering care in South Australia,^25^ LTCF providers are now provided individualized reports on their performance, with benchmarks to inform quality improvement.^38^ Furthermore, with a new model of onsite pharmacist practice recently implemented in Australian LTCFs at a cost of AUD 350 million over 4 years, we have commenced funded work to develop, validate, implement, cost, and disseminate the Pharmacists Actioning Rational Use of Medicines in Aged Care (PHARMA-Care) quality monitoring program. The PHARMA-Care program will provide a quality framework for monitoring medication management and pharmacist services in Australian LTCFs and develop quality measures that leverage routinely collected data from ROSA and eMM systems, to minimize the burden of data collection for care providers.

## Considerations when using real-world data to produce valid and impactful evidence for decision-makers

Based on our experiences, we suggest considering the following points when designing, conducting, and disseminating results of medication safety and effectiveness studies specific to LTCF settings. First, considerable planning and resources are required to manage the data access and ethical approvals for analyzing integrated data sets required to examine cross-sectoral care and outcomes (eg, aged care and health care). ROSA, for example, comprises 17 data sources from 9 data custodians and requires 5 authorized data integration authorities to link its data sources. Its data sources are underpinned by more than 30 separate ethical and governance approvals.

Second, individuals entering LTCFs are increasingly older and have greater multimorbidity,^11^ necessitating careful selection of study periods and adjustment for covariates such as age, sex, comorbidities, and year of study entry. Residents often have complex pathways to LTCF entry (ie, involving hospitalization or respite/temporary care) and individuals may receive care from a new GP or care provider at facility entry.^39^ We have observed considerable variability in medication use at LTCF entry, likely resulting from changes in medication regimens and the redispensing of medications at hospital discharge or LTCF entry.^18^^,^^26^ Stratifying study cohorts by time since LTCF entry may be necessary to comprehensively examine associations between interventions and changes in medication use or health outcomes, with inclusion of periods when medication and health care use have stabilized after entry.^18^^,^^35^

Third, establishing a cohort for an active comparator, new user study design^40^ can be challenging in LTCF populations, especially when examining safety and effectiveness of newly marketed medications, because chronic conditions may have been treated for lengthy periods prior to admission. Comparison groups may also have differences in health conditions or disease severity that may bias estimates.

Fourth, defined daily doses represent the usual adult dose for a medication’s main indication but may not always represent doses used by residents.^41^ Medication exposure may need to be ascertained using a range of measures, such as prevalence, defined daily doses, and standard doses for frail older people.

Fifth, the median length of a long-term care episode in Australian LTCFs in 2022-23 was 20.5 months and 84% of residents exited due to death,^42^ with 10% of residents dying within 3 months of LTCF entry.^43^ Residents with increased frailty or certain health conditions such as dementia may be more or less likely to receive certain interventions or models of care. Robust approaches to mitigate risk of survivor bias, adjust for confounders (eg, propensity score approaches), and account for competing events are required.

Last, involving consumers and end users in study design, interpretation, and dissemination activities is an international priority area and aids translation of evidence into practice.^44^ We have successfully established consumer advisory committees and engagement strategies, included consumers as co-investigators, partnered early-career researchers with consumer “mentors,” co-presented with consumers at public events, and co-produced media releases and infographics to embed user perspectives.

## Concluding remarks

Various sources of real-world data are available to conduct pharmacoepidemiological studies in the long-term care setting, where a strong evidence base for optimal medication use is generally lacking. Examples discussed in this commentary highlight the importance of real-world data and robust methodological approaches to generate timely, real-world evidence required to inform LTCF policy and practice. The complex nature of medication management in older people, together with continued long-term care reforms worldwide and challenges with conducting RCTs in LTCFs, means there is ongoing need for real-world evidence about medication safety and effectiveness among residents of LTCFs that pharmacoepidemiologists can help to address.

## Acknowledgments

We acknowledge the ROSA Steering Committee and the ROSA South Australian Health and Medical Research Institute (SAHMRI) Research Team for ensuring the success of the ROSA and support of the work discussed in this commentary. We also acknowledge the South Australian Government Department for Innovation and Skills (2017-2021), which provided us with support to establish ROSA; the Australian Government Medical Research Future Fund (2021-2024, PHRDI000009); and ROSA collaborating partners (SAHMRI; ECH Inc.; Silver Chain; Life Care) for ongoing support, and the Australian Institute of Health and Welfare for the linkage and construction of input data.
